# Occurrence of High Microsatellite-Instability/Mismatch Repair Deficiency in Nearly 2,000 Human Adenocarcinomas of the Gastrointestinal Tract, Pancreas, and Bile Ducts: A Study From a Large German Comprehensive Cancer Center

**DOI:** 10.3389/fonc.2021.569475

**Published:** 2021-07-22

**Authors:** Alexander Quaas, Jan Rehkaemper, Josef Rueschoff, Aylin Pamuk, Thomas Zander, Axel Hillmer, Janna Siemanowski, Jana Wittig, Reinhard Buettner, Patrick Plum, Felix Popp, Florian Gebauer, Christiane Josephine Bruns, Heike Loeser, Hakan Alakus, Birgid Schoemig-Markiefka

**Affiliations:** ^1^ Institute of Pathology, University Hospital Cologne, Cologne, Germany; ^2^ Institute of Pathology, Nordhessen and Targos Molecular Pathology GmbH, Kassel, Germany; ^3^ Department of General, Visceral and Cancer Surgery, University Hospital Cologne, Cologne, Germany; ^4^ Department of Internal Medicine I, University Hospital Cologne, Cologne, Germany

**Keywords:** Union for International Cancer Control (UICC) stage 4, esophageal adenocarcinoma, gastric carcinoma, high microsatellite-instability (MSI-H), microsatellite-instability

## Abstract

**Introduction:**

Knowledge of the high microsatellite-instability (MSI-H)/mismatch repair deficiency (MMRd) status is of increasing interest for personalized neoadjuvant or adjuvant therapy planning. Only a few studies are available on MSI-H distribution in the Northern European Caucasian patient population. In this study, we focused on a large cohort of tumors of the upper gastrointestinal tract.

**Materials and Methods:**

Surgical material from a total of 1,965 patients was analyzed for MSI-H/MMRd status (including 1,267 carcinomas of the esophagus or stomach). All tumors were analyzed with an internationally recommended immunohistochemical panel consisting of four antibodies (MLH1, MSH2, PMS2, and MSH6). The results were molecularly objectified.

**Results:**

Adenocarcinomas with MSI-H/MMRd were detected with the following distribution: esophagus (1.4%), stomach (8.3%), small intestine (18.2%), large intestine (8.5%), intrahepatic bile ducts (1.9%), and pancreas (0%). In case of gastric tumors with MSI-H/MMRd, neoadjuvant therapy did not influence the prognosis of patients (p = 0.94). Within all tumor entities with MSI-H/MMRd, patients with a UICC stage 4 were also represented. In this advanced stage, 11.7% of patients with MSS tumors were diagnosed compared to 0.5% of patients with MSI-H tumors relative to the entire tumor collective.

**Discussion:**

In this study, the proportion of MSI-H/MMRd tumors in the stomach is smaller than would have been expected in knowledge of the data published by TCGA or AGRC. Negative prognostic effects regarding MSI-H status and neoadjuvant therapy as described by the MAGIC study group were not seen in our cohort. The extent to which the MSI-H/MMRd status should be known for neoadjuvant therapy planning must be clarified in prospective studies in the future. At present, there is no convincing data to dispense the neoadjuvant therapy for gastric carcinoma. Due to the very convincing, positive data regarding the response rates of MSI-H tumors to treatment with PD1/PD-L1 inhibitors, every metastatic carcinoma of the gastrointestinal tract should be tested for its MSI-H status.

## Introduction

Over the last years, an increasing number of tumor entities were analyzed for high microsatellite-instability (MSI-H)/mismatch repair deficiency (MMRd) (1). MSI is characterized by tumor DNA sequence alterations, namely deletions or expansion of short tandem repeats (mainly mono- or dinucleotide motifs) in microsatellite regions in comparison to non-tumor DNA. These DNA alterations accumulate because of a failure of the DNA mismatch repair (MMR) system that normally repairs errors occurring during DNA replication (2). MMRd leads to an increased accumulation of DNA alterations genome wide, which are assumed indirectly by testing for MSI. The underlying pathogenic mechanisms are hypermethylation of the promotor regions of genes encoding MMR proteins in most cases or mutations in MMR genes (*e.g.*, MLH-1, PMS-2, MSH-2, and MSH-6) resulting in loss of function of the entire MMR system. It is proposed that immunohistochemistry that takes into account four proteins (MLH1, MSH2, MSH6, and PMS2) is sufficient as a screening method for a MSI phenotype in diagnostic algorithms. If these four proteins are detected by immunohistochemistry in the tumor cell nuclei, no further testing is necessary as the tumor is assumed to harbor no MMRd. These tumors are often classified as “microsatellite stable (MSS),” as no MSI is assumed in the presence of expressed MMR proteins [exceptions exist with very rare forms of Lynch syndrome (LS)] (3–6).

The MMR proteins form a functional complex consisting of two heterodimers MLH1–PMS2 and MSH2–MSH6.

The PMS2 protein degenerates in the absence of MLH1 (therefore, MLH1 promoter methylation or mutation in *MLH1* occurs, leading to loss of function of MLH1, also leads to missing the detection of PMS2 in immunohistochemistry), and MSH6 degenerates in the absence of MSH2. However, MLH1 and MSH2 proteins can remain stable on their own without their respective partners. Loss of nuclear staining in the tumor cells either with MLH1/PMS2 or MSH2/MSH6 constitutes a MSI-H phenotype. Mutations in MMR genes are known to occur and are classified as either sporadic or less frequently hereditary, with the latter defining LS (formerly HNPCC). Because almost all tumors with mutations in the MMR genes display the MSI phenotype, MSI testing has become a useful diagnostic tool in screening for cancer predisposing to LS (7, 8).

More recently, studies have focused on the prognostic and predictive value of the MSI phenotype independent of the underlying repair defect. For example, colorectal carcinomas (CRCs) with a MSI phenotype were reported to display less frequent distant metastasis in comparison to that in MSS tumors, and patients had a more favorable outcome (9, 10).

Furthermore, the MSI phenotype of tumors was shown to be a predictive marker for a more sensitive response to immune checkpoint blockade (*e.g.*, anti-PD-1 inhibitor) with improved clinical benefit (11). It is assumed that the large amount of frameshift-derived neoantigens resulting from MMRd (sporadic and hereditary) leads to a more sensitive response to immune checkpoint blockade, regardless of specific cancer entity (12).

Little reliable data exist on the incidence of MSI-H tumors of the upper GI tract in the Northern European Caucasian patient population. While there are several publications on CRC, carcinomas of the upper gastrointestinal tract or bile ducts in particular are underrepresented and often considered only in a very small number of patients. Therefore, the aim of this study was to determine the incidence of the MSI-H phenotype in a Northern European Caucasian patient cohort, considering nearly 2,000 unselected human gastrointestinal or biliodigestive carcinomas, with a special focus on the poorly characterized carcinoma entities of the upper gastrointestinal tract.

## Materials and Methods

### Statistical Analysis

Patient data were prospectively collected in a database. Overall survival was evaluated from the date of surgery to death. Kaplan–Meier curves were generated and compared using the log-rank test. Data on patients with no event or lost follow-up were censored at the last date of consultation. A two-sided p-value < 0.05 was considered statistically significant. SPSS package version 25 (IBM, Armonk, New York, USA) was used for all statistical analyses.

### Patients and Tumor Samples

In this retrospective study, we analyzed formalin-fixed and paraffin-embedded human tumor tissue for its DNA MMR protein status/microsatellite status. For this purpose, surgical material from different tumor entities was considered. We included all patients who underwent surgery in our Cancer Centers (with some exceptions of the pancreatic tumor collective and two tumors of the small intestine) during a 15-year period. We had access to the tumor material within the selected time period. There was no pre-selection of patients beyond that. In total, 1,965 carcinomas were analyzed (**Table 1**). Representative tumor material was transferred to tissue microarrays (TMAs) for immunohistochemical analysis. To determine the DNA repair protein status, four proteins (MLH1, PMS2, MSH2, and MSH6) were immunohistochemically determined. Additionally, we examined all CRCs for their expression of MSH3 to determine the extent of “elevated microsatellite alterations at selected tetranucleotide repeats” (EMASTs) in our collective. Thus, in a first step all tumors were primarily screened for MMR status by immunohistochemistry. A tumor was considered mismatch-repair deficient (MMR-d) if two contiguous protein pairs (MLH1/PMS2 or MSH6/MSH2) or MSH3 in CRCs) showed a nuclear protein loss in the tumor cells, while the surrounding non-tumor cells (inflammatory cells or fibroblasts) showed preserved nuclear staining (positive internal control). We did not observe an isolated protein failure, which can occur in connection with LS, in our tumor collective. All cases with abnormalities of the staining pattern were re-analyzed on large tumor blocks in order to verify the result of the TMA evaluation and to be able to make statements regarding a possible heterogeneous distribution of DNA repair protein-deficient tumor clones. Nevertheless, we verified the results on a molecular level in exemplary cases using an in-house PCR additionally (see details below). With this approach, there was only one gastric carcinoma where we were unable to show MSI-H, although we detected a focal failure of MSH6 protein expression with unknown significance in a few hundred tumor cells. We were able to show the concordance between the immunohistochemical and molecular results in all other analyzed cases. As a result, we restricted ourselves to protein analysis for the remaining tumors.

**Table 1:** Characteristics of the patients.

	EAC	GAC	SBAC	LBAC	PDAC	BDAC
n (total)	685	582	11	319	316	52
n (MSI-H)	8 (1.4%)	44 (8.3%)	2 (18.2%)	27 (8.5%)	0 (0%)	1 (1.9%)
**UICC Stage**						
1	144	90	1	49	18	10
2	166	110	1	78	118	14
3	289	127	5	34	8	4
4	78	63	4	79	15	24
not available	8	138	0	79	0	0
**Gender**						
female	85	166	3	120	146	21
male	600	327	8	199	170	31
not available	0	35	0	0	0	0
**Age**						
>60	349	132	4	250	236	13
<60	305	286	6	69	78	39
not available	31	110	1	0	0	0

Compilation of all examined patients and the tumor characteristics (UICC tumor stages, gender and age distribution). EAC, esophageal adenocarcinoma; GAC, gastric adenocarcinoma; SBAC, small bowel adenocarcinoma; LBAC, large bowel adenocarcinoma; PDAC, pancreatic ductal adenocarcinoma; BDAC, bile duct adenocarcinoma.

### Ethics Committee Approval

Procedures were followed as outlined in accordance with ethical standards formulated in the Helsiniki Declaration 1975 (and revised in 1983). Patients gave their written consent to usage of their tumor specimens, and the objective of the projects is primarily in the field of diagnostic and quality assurance. An approval was obtained from the University of Cologne Ethics Committee (reference number: 13-091 and 10-242).

### TMA Construction

For TMAs, one tissue core from each tumor was punched out and transferred into a TMA recipient block. TMA construction was performed as previously described (13, 14). In brief, tissue cylinders with a diameter of 1.2 mm each were punched from selected tumor tissue blocks using a self-constructed semi-automated precision instrument and embedded in empty recipient paraffin blocks, and 4 μm sections of the resulting TMA blocks or large scale tumor blocks were transferred to an adhesive coated slide system (Instrumedics Inc., Hackensack, NJ, USA) for immunohistochemistry.

### Immunohistochemical Analysis of MMR Proteins

All tumors were stained for MLH1 (clone: M1 Ventana), MSH2 (G219-1129), PMS2 (EPR3947), and MSH6 (Clone44, Ventana) on Ventana Benchmark stainers. Additionally, all CRCs were stained for MSH3 (clone EPR 4334) on the Bond stainer from Leica, Germany, using EDTA buffer (1:100 dilution). 3,3′-Diaminobenzidine (DAB) was used as a chromogen and hematoxylin as a counterstain.

### Molecular Analysis

Tumor areas were marked by an experienced pathologist on an H&E stained slide and corresponding unstained tumor, and paired normal tissues were macrodissected from formalin-fixed, paraffin-embedded (FFPE) 10 µm thick tissue sections. DNA extraction was performed with the Maxwell 16 FFPE Plus Tissue LEV DNA Purification Kit (Promega, Mannheim, Germany) on the Maxwell 16 (Promega, Mannheim, Germany) following the instructions of the manufacturer after overnight digestion with Proteinase K.

Microsatellite status was determined using an in-house PCR protocol with primers for the Bethesda markers, including the mononucleotide markers BAT25 and BAT26 or the dinucleotide markers D5S346, D2S123, D17S250, D10S197, D18S58, and D13S153 and the tetranucleotide marker MYCL1. For evaluation, polymerase chain reaction (PCR) was followed by fragment length analysis on an ABI PRISM 3500 Genetic Analyzer (Applied Biosystems, Life Technologies, Darmstadt, Germany).

## Results

**Figure 1:**
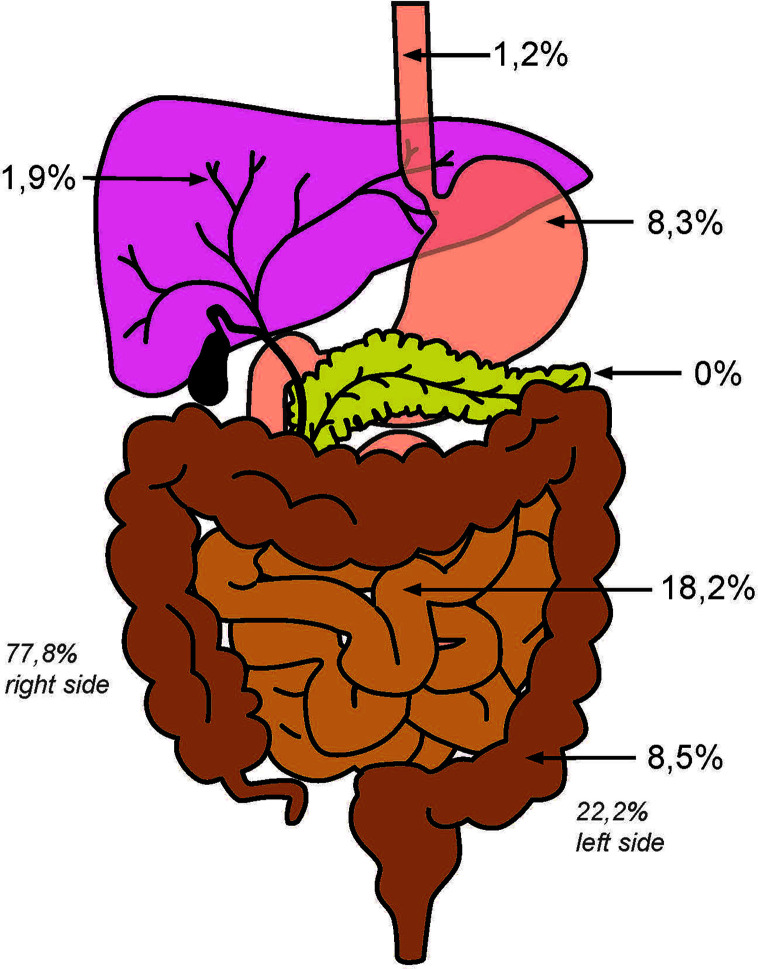
Distribution of MSI-H carcinomas in the GI-tract.

In the present study, we analyzed the MMRd status of 1,965 adenocarcinomas from the gastrointestinal tract and biliopancreatic system. In 82 out of 1,965 cases (4.2%), we observed a loss of expression at the protein level in the tumor cells of at least one of the two pairs of MMR proteins (MLH1/PMS2 or MSH6/MSH2). These tumor tissues were then tested for MSI-H by an in-house PCR protocol (as described above). The results confirmed the concordant MSI-H/MMRd status of the exemplary molecularly tested tumors (compare **Figure 1**). For available clinical patient characteristics, see **Tables 1** **–** **3**.

### UICC Stage 4 in MSS and MSI Tumors Related to All Tumor Entities

Within the whole patient cohort, in this study, 229 MSS tumors presented in our operative tumor collective with UICC stage 4 (11.7%) compared to 10 tumors with MSI (0.5%). In the MSI subgroup, 12.2% of patients were diagnosed with UICC stage 4 (**Table 2**).

**Table 2:** Characteristics of MSI-H tumors.

	EAC	GAC	SBAC	LBAC	PDAC	BDAC
n (total)	8	44	2	27	0	1
**UICC Stage**						
1	0	13	0	7	0	0
2	2	10	0	10	0	0
3	5	13	3	5	0	0
4	1	3	0	5	0	1
**Gender**						
female	2	14	0	15	0	0
male	6	25	2	12	0	1
**Age**						
>60	3	38	1	25	0	1
<60	5	6	1	2	0	0

Compilation of tumor and patient characteristics with MSI-H (UICC tumor stages, gender and age distribution). EAC, esophageal adenocarcinoma; GAC, gastric adenocarcinoma; SBAC, small bowel adenocarcinoma; LBAC, large bowel adenocarcinoma; PDAC, pancreatic ductal adenocarcinoma; BDAC, bile duct adenocarcinoma.

### Esophageal Adenocarcinoma

Out of 685 patients with EAC, 562 patients were finally analyzable. In eight out of 562 (1.4%) cases of EAC, the analysis revealed MSI-H tumors. All of these tumors showed a loss of MLH1 and PMS2 at the protein level. Patient characteristics are given in **Tables 1**, **2**. Reasons for non-informative cases included lack of tissue samples or absence of unequivocal cancer tissue in the TMA spot. Not all clinical data were available from the 31 patients. This is in line with the idea that EACs are predominantly assigned to the chromosomally instable (CIN) subgroup of gastric carcinoma (compare *Discussion*) (15).

In the subgroup of MSI-H tumors, seven out of eight tumors were localized with lymph nodal metastasis (UICC stage 2 or 3). One patient presented with an oligometastasized UICC stage 4 tumor.

### Gastric Adenocarcinoma

**Figure 2:**
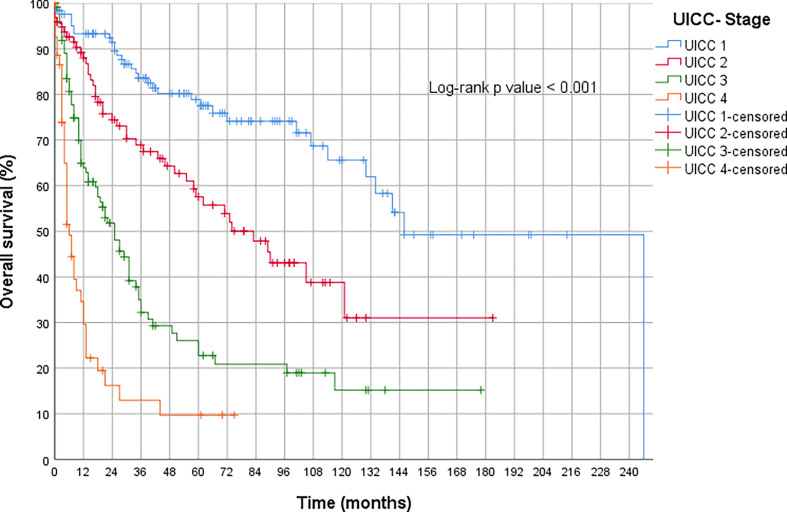
Overall survival of all primary operated gastric carcinomas.

The subgroup of GACs comprises 582 patients, of which 528 were analyzable. Of these, 44 tumors were classified as MSI-H (8.3%), significantly less than we expected according to TCGA (compare *Discussion*). In order to check the quality of our collective, we have presented the outstanding prognostic importance of UICC staging (compare **Figure 2**).

In the MSI-H subgroup, 28 patients underwent primary surgical resection without neoadjuvant therapy, while 11 patients received neoadjuvant therapy with cytostatic combinations. The majority of cases were diagnosed in either a localized or less advanced tumor stage (UICC stages 1 and 2; n = 23, 58%). Nevertheless, 19 patients already harbored regional lymph node metastases (48.1%) at time of diagnosis (**Table 3**). Three patients presented with distant metastases already at the time of diagnosis or shortly after surgery (UICC stage 4; 7.6%).

**Table 3:** Characteristics of MSI-H gastric carcinomas.

	GAC primary resected	GAC neoadjuvant treated	total	p-value
n (MSI-H) = 44	28	11	39	
**UICC Stage**				
1	10 (35.7%)	3 (27.3%)	39	0.066
2	9 (32.1%)	1 (9.1%)		
3	6 (21.4%)	7 (63.6%)		
4	3 (10.7%)	0 (0%)		
**Gender**				
female	8 (28.6%)	6 (54.5%)	39	0.156
male	20 (71.4%)	5 (45.5%)		
**Age**				
>60	24 (88.9%)	8 (72.7%)	38	0.329
<60	3 (11.1%)	3 (27.3%)		
**Localization**				
gastro-esophageal junction	6 (23.1%)	4 (36.4%)	37	0.812
proximal	3 (11.5%)	2 (18.2%)		
mid-body	4 (15.4%)	1 (9.1%)		
distal	10 (38.5%)	4 (36.4%)		
stomach	1 (3.8%)	0		
anastomosis	2 (7.7%)	0		
**pT**				
1	7 (25.0%)	2 (18.2%)	39	0.151
2	9 (32.1%)	2 (18.2%)		
3	10 (35.7%)	3 (27.3%)		
4	2 (7.1%)	4 (36.4%)		
**pN**				
0	15 (53.6%)	5 (45.5%)	39	0.723
1	7 (25.0%)	4 (36.4%)		
2	5 (17.9%)	1 (9.1%)		
3	1 (3.6%)	1 (9.1%)		

This table summarizes the gastric carcinomas and their characteristics, which show a high microsatellite instability (MSI-H). GAC, gastric adenocarcinoma.

**Figure 3:**
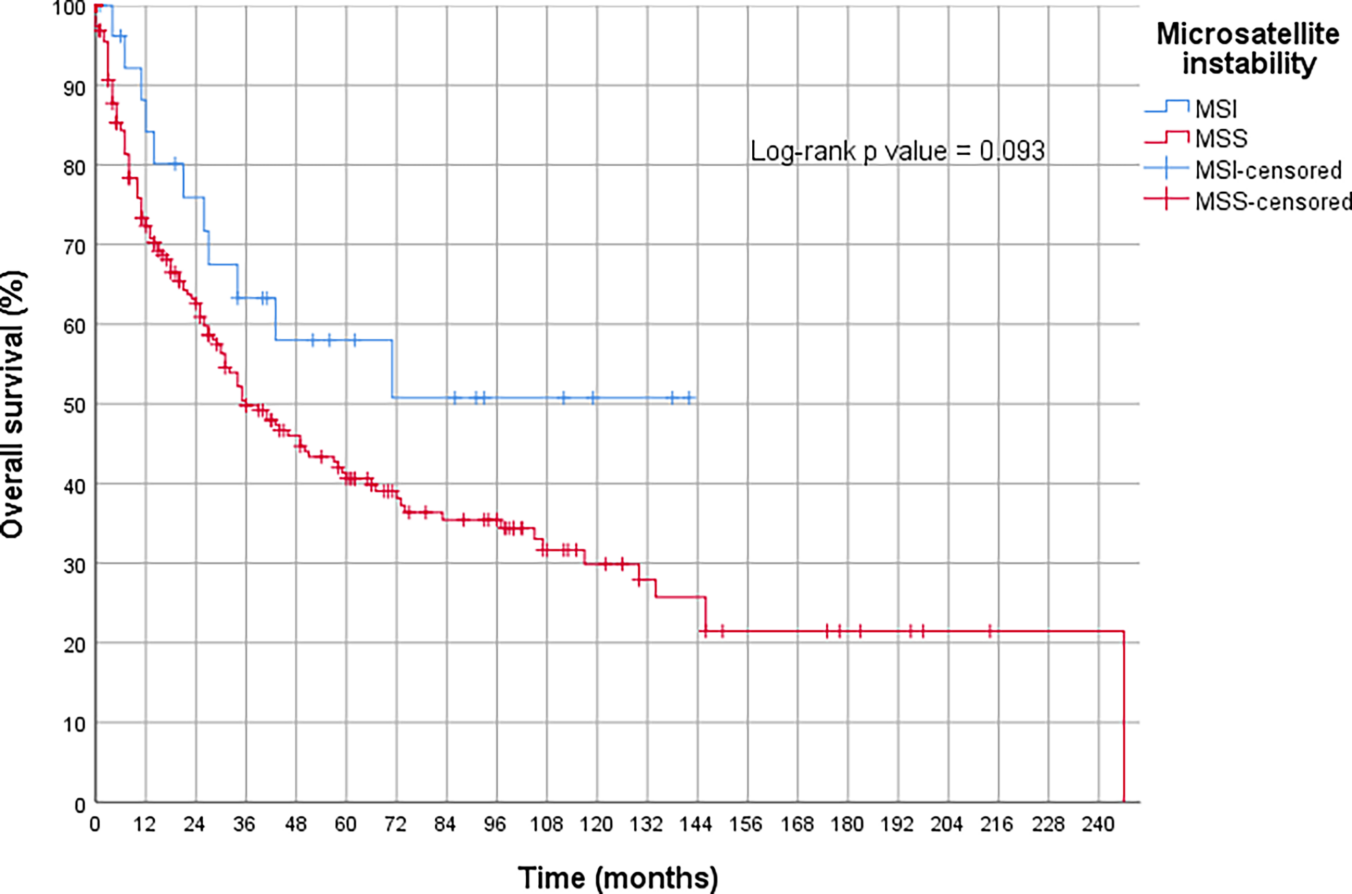
Overall survival in gastric cancer with high microsatellite instability (MSI-H) without neoadjuvant treatment compared to microsatellite stable tumors (MSS).

**Figure 4:**
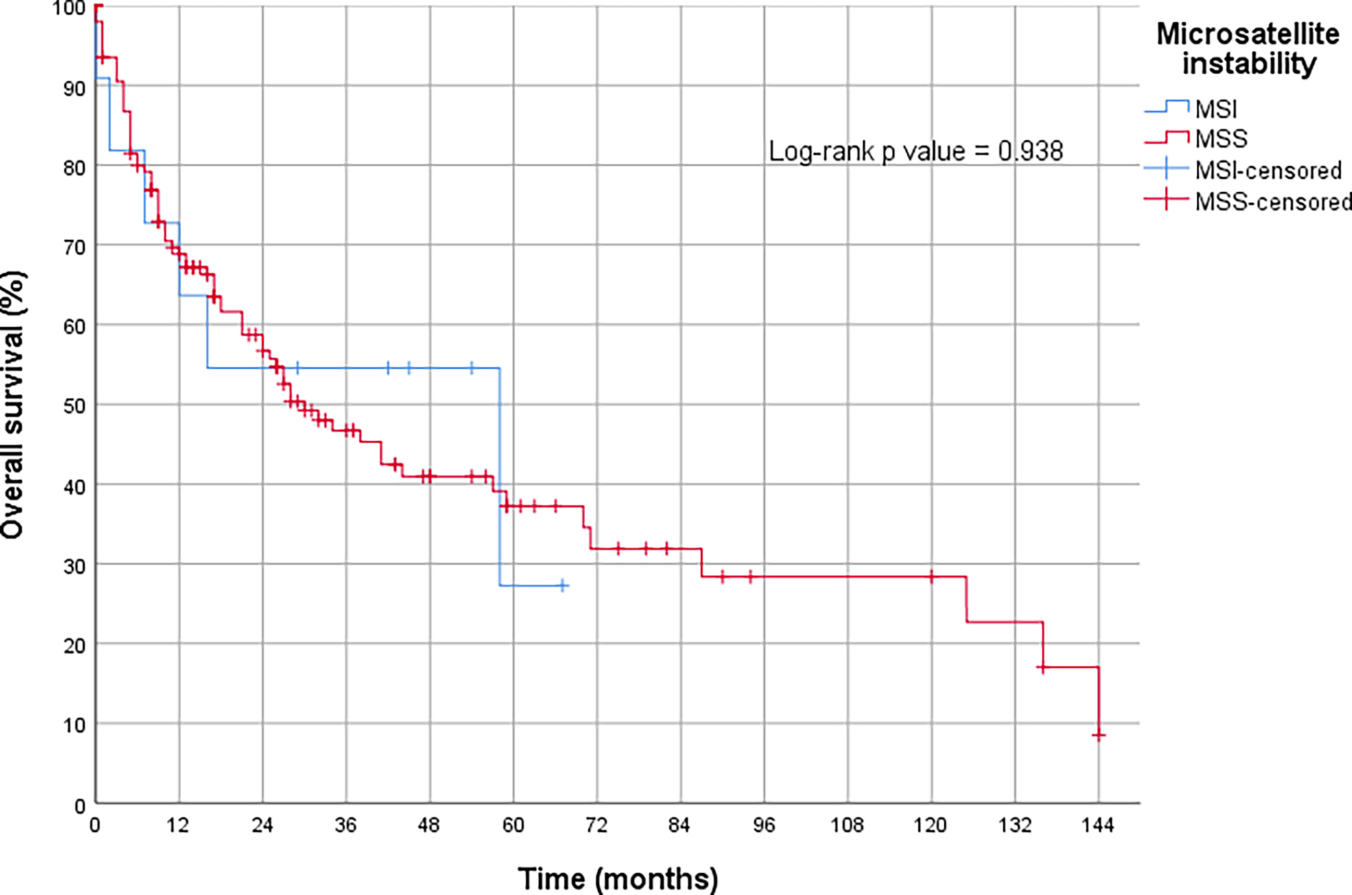
Overall survival in gastric cancer with high microsatellite instability (MSI-H) after neoadjuvant treatment.

The OS analysis revealed that the MSI-H tumors treated with primary surgery tended to show a better prognosis compared to the MSS tumors treated the same way. However, this result was only slightly insignificant (p = 0.09) (compare **Figure 3**). Neoadjuvant therapy had no measurable influence on the prognosis of MSI-H tumors (p = 0.94) in our collective (**Figure 4** and **Table 3**).

All MSI-H gastric carcinomas were examined for possible heterogeneity of MMR protein expression. For this purpose, all tumor-bearing paraffin blocks with the corresponding precipitated protein were re-analyzed. No heterogeneous expression was found that would have been suspicious for MSS clones within a MSI-H tumor. Also, the lymph node metastases showed a homogeneous MSI-H phenotype.

### Small Bowel Adenocarcinoma

Within the SBAC subgroup (n = 11), two MSI-H carcinomas were detected (18.2%). One tumor occurred in association with LS. However, both MSI-H tumors presented in a locally advanced, regional lympho-nodal metastatic stage (UICC stage 3).

### Large Bowel Adenocarcinoma

All 319 LBAC tumors included were analyzable. In 27 cases (8.5%), a MSI-H phenotype was revealed, which was more frequently localized in the right colon (n = 21; 77.8%). Two tumors occurred in association with LS. From 240 patients, we have additional clinical follow-up data. In the overall cohort, almost twice as many men received surgery (199 men *versus* 120 women). However, in the MSI-H group, there were more women (15 women *versus* 12 men). While in the overall cohort UICC stages 1 and 2 are represented with 127 patients; 113 patients were diagnosed in advanced tumor stages 3 and 4. Thus, both UICC groups (1 + 2 *versus* 3 + 4) are almost equally distributed. The metastatic UICC stage 4 is especially strongly represented with 79 patients (24.8%). In the MSI-H group, an enrichment of UICC stages 1 and 2 (n = 10; 37%) was found. Of 79 patients who presented with UICC stage 4, five patients had MSI-H tumors (6.3%; 18.5% UICC stage 4 tumors in the MSI-H subgroup). Additionally, we found a single patient with loss of MSH3 and confirmed instability of the tetranucleotide MYCL1. In our collective of CRCs, we found a frequency of 0.3% for EMAST. Further patient characteristics are given in **Tables 1**, **2**.

### Bile Duct Adenocarcinoma

Intrahepatic bile duct carcinomas of 52 patients were available for analysis. Clinical data were available for all patients. One tumor showed MSI with selective loss of MLH1 and PMS2 (1.9%). This male patient was over 60 years old and presented clinically with advanced UICC stage 4 (**Tables 1**, **2**).

### Pancreatic Ductal Adenocarcinoma

A total of 319 PDAC tumors were analyzed. All tested carcinomas showed a MSS phenotype. Clinical data was available for all patients. Patient characteristics are given in **Tables 1**, **2**.

## Discussion

MSI-H is a predictor for the probability of an increased response to immune checkpoint inhibitors directed against PD-1/PD-L1 in different carcinoma entities (11, 12, 16). At the same time, MSI-H tumors do not seem to benefit from cytostatic therapy with 5-FU. This may be due to the fact that chemosensitivity requires the integration of 5-FU into tumor DNA and that an intact MMR system is required for this integration (17, 18). The prognostic relevance of a neoadjuvant therapy for patients with MSI-H gastric cancer is unclear. While one study showed an unfavorable prognosis, indicating that neoadjuvant chemotherapy should not be used in this setting, other studies found no such effect (19–21). Knowledge of the MSI status of the tumor is therefore of increasing interest for therapy planning, irrespective of the question of possible LS. Two procedures are recommended to determine the MSI status. Internationally, an immunohistochemical analytical method, including the four MMR proteins mentioned above, is recommended as a screening method. Alternatively or in addition, a PCR- or NGS-based method can be used (8, 22–24). Both methods were used in this study to determine the MSI status of the tumors analyzed here.

Over the last 5 years, several publications have described the extent of expected MSI-H tumors in different tumor entities (1, 25). The majority of studies did not focus on Northern European patients. There is no clarity about the extent to which the therapy-relevant subgroup of MSI-H tumors is represented in the German patient population and what differences arise between this subgroup and Southern European or non-European populations. We have therefore analyzed more than 1,900 tumors of the upper or lower gastrointestinal tract, pancreas or intrahepatic bile ducts for their MSI-H status. The tumor material originated mainly from a single large German surgical center. In accordance with the data of other research groups, there are about 1% of MSI-H tumors that are EACs (26–28). To the best of our knowledge, there are nine publications to date that deal with the frequency of MMRd in EAC. Including the data of the TCGA group (n = 70), a total of 285 adenocarcinomas were analyzed in these studies (n = 5 to n = 70). Only a proportion of these patients were of Caucasian origin. The extent of MSI-H varied between 0 and 20%, with 20% in a small study referring to only one patient. The 70 EACs analyzed from the TCGA group were all MSS (26–35). Thus, LS as a hereditary cause of MSI-H does not play a relevant role in the pathogenesis of esophageal cancer. The very low frequency of MSI-H in esophageal cancer will also allow conclusions to be drawn about the response rates to immune checkpoint inhibitor treatment.

Adenocarcinomas of the stomach show significantly more MSI-H tumors compared to the esophagus. Considering TCGA, up to 21% of gastric carcinomas were MSI-H (36, 37). Nevertheless, other data on the MSI-H frequency in gastric cancer is highly variable and fluctuates between 7 and 24% (25). Recently, a study was published that molecularly characterized more than 600 adenocarcinomas of the stomach and the gastroesophageal junction. It is assumed that this group studied a Northern European Caucasian patient population. The well-documented analysis results show practically identical results to our tumor cohort, with 9.6% MSI-H in surgically removed gastric cancers (38). If the data of this group and our data are combined, it can be assumed that less than 10% of MSI-H gastric carcinomas in Germany have been diagnosed within over 1,000 tested gastric carcinomas. This information is particularly relevant in view of the good response rates to immune checkpoint therapies in this subtype, as the MSI-H subtype is to be expected in less than half of German patients with gastric cancer compared to that of TCGA data (MSI-H in TCGA collective 21.9%). According to our results, metastasized MSI-H tumors (UICC stage 4) are very rare, at least in a potentially operable patient collective. However, this statement is strongly flawed, since we have only analyzed surgical material, and gastrectomy is usually not performed on already hematogenously metastasized gastric carcinomas. Reliable statements about the frequency of MSI-H phenotype in the (advanced) hematogenously metastasized state (UICC stage 4) are not possible. Although, since the MSI-H status is associated with a favorable prognosis in all studies, a relevant number of patients with UICC stage 4 MSI-H cannot be assumed. As mentioned above, contradictory data exist regarding the prognostic relevance of neoadjuvant chemotherapy in patients with MSI-H gastric cancer. We did not find any statistical significance (see also **Figure 3**). In our collective, there was neither a less beneficial prognosis in the MSI-H neoadjuvant treated group as suggested by the retrospective data of the MAGIC study group nor a favorable prognosis as demonstrated in the Heidelberger study group. In the future, larger and prospectively considered patient collectives will have to clarify the prognostic and predictive effects of neoadjuvant therapy in the MSI-H group. From our point of view, the contradictory data available to date do not justify abandoning neoadjuvant treatment in MSI-H gastric carcinoma (38).

The proportion of MSI-H colon carcinomas in our collective with 8.5% is slightly below the internationally usual proportion of about 13–15%. We explain this by the fact that the sporadic MSI-H tumors of the colon occur in older patients who are under-represented in our university hospital (39, 40). The proportion of UICC stage 4 colon cancer in our patient population is higher than expected. This is possibly explained by the university structure of visceral surgery with its own hepatobiliary unit, possibly leading to an accentuation of patients in advanced tumor stages.

This underlines two characteristics and also possible weaknesses of this publication. First, it is a retrospective analysis of a large but solitary university cancer center. Therefore, the patient population is biased. Second, the main focus of the Visceral Surgery Clinic is the treatment of carcinomas of the upper gastrointestinal tract; for these tumor entities, the center and this publication will probably be able to provide very realistic and representative data on the MSI-H distribution in the surgical material. This is limited for carcinomas of the lower gastrointestinal tract for the reasons described above. A further weakness lies in the sole analysis of the surgically removed tumor material considered by us. Most patients showed no distant metastases at the time of surgery. In the remaining cases, the maximum was that of resectable metastases in an oligometastatic fashion. Patients who at the time of diagnosis were either intensively metastasized or were not operable for other reasons were not considered. It is not to be assumed and does not correspond to the clinical experience of the authors that a quantitatively significant proportion of MSI-H tumors occur in the group of patients with extensive distant metastases. At least in the group that received surgery, patients with oligometastasized gastric carcinoma, a comparably high proportion of MSI-H tumors were found as in the rest of all gastric tumors. However, our data also make it clear that MSI-H tumors are also found in a metastatic tumor stage despite their otherwise more favorable prognosis. The authors of this study argue that at least all metastatic GACs should be analyzed for their MSI-H status.

Molecular analyses on hundreds of adenocarcinomas revealed a comparable number of MSI-H tumors in the small intestine and colon (41, 42).

Additionally, we can confirm the rare sequence for the MSI-H bile duct carcinoma in our collective. In the prognostically and therapeutically extremely unfavorable ductal adenocarcinoma of the pancreas, we did not find any MSI-H tumors in our patient collective. Nevertheless, other major analyses rarely describe MSI-H tumors in the pancreas as well. However, the expected frequency is below 1%.

In summary, MSI-H is also distributed with varying frequency to a Northern European patient population within the gastrointestinal tract. In the esophagus, MSI-H tumors are very rare (about 1%). In the stomach, they represent a subgroup of about 10%. The proportion of MSI-H tumors in the stomach is therefore significantly smaller than would have been expected after TCGA or AGRC. We did not see any negative prognostic effects regarding the MSI-H status and neoadjuvant therapy as described by the MAGIC study group. The extent to which the MSI-H status should be known for neoadjuvant therapy planning must be clarified in prospective studies in the future. At this point in time, there is no convincing data to dispense with neoadjuvant therapy for gastric carcinoma. MSI-H tumors also occur in metastasized UICC stage 4 tumors. Due to the very convincing, positive data regarding the response rates of MSI-H tumors to treatment with PD1/PD-L1 inhibitors, every metastatic carcinoma of the gastrointestinal tract should be tested for its MSI-H status, independently of the question of underlying LS.

## Data Availability Statement

The raw data supporting the conclusions of this article will be made available by the authors, without undue reservation.

## Ethics Statement

The studies involving human participants were reviewed and approved by the University of Cologne Ethics Committee (reference number: 13–091 and 10–242). The patients/participants provided their written informed consent to participate in this study.

## Author Contributions

AQ, JRe, HA, and BS-M made substantial contributions to conception and design. HA, AQ, JRu, CB, RB, AH, AP, TZ, JS, JW, PP, FP, FG, HL, BS-M, and JRe were responsible for acquisition of data. AQ, JRe, MK, and HA were responsible for analyses and interpretation of data. All authors contributed to the article and approved the submitted version.

## Conflict of Interest

Author JR was employed by company Nordhessen and Targos Molecular Pathology GmbH.

The remaining authors declare that the research was conducted in the absence of any commercial or financial relationships that could be construed as a potential conflict of interest.
